# The American Women's War Hospital

**Published:** 1915-02-20

**Authors:** Mary Harcourt

**Affiliations:** Hon. Secretary. 31 Old Burlington Street, London, W.


### THE EDITOR'S LETTER-BOX.

### The American Women's War Hospital.

*To the Editor of* The Hospital.

Sir,—We have received a cutting from the last issue of your charming paper, The Hospital, giving a most kindly notice of the American Women's War Hospital at Paignton, for which many thanks. One sentence of the notice, however, is somewhat incorrect, in that it states that the "hospital is entirely staffed by Americans." May I point out that this is not so, and my committee would be very sorry indeed if such an impression arose in this country?

There are now at the hospital the two American units so generously sent from America, but the matron is English, and the original staff of English nurses (two, I think, of the *really* original ones have left) is still there, and we hope will continue to be, so that all the wards of the hospital are now staffed, as always, by both American and English representatives.—Yours truly,

Mary Harcourt, Hon. Secretary.

31 Old Burlington Street, London, W.
February 15, 1915.

### The Clinical Value of Poor-Law Infirmaries.

*To the Editor of* The Hospital.

